# Identification of Gender Differences in Acute Myocardial Infarction Presentation and Management at Aga Khan University Hospital-Pakistan: Natural Language Processing Application in a Dataset of Patients With Cardiovascular Disease

**DOI:** 10.2196/42774

**Published:** 2024-12-20

**Authors:** Christine Ngaruiya, Zainab Samad, Salma Tajuddin, Zarmeen Nasim, Rebecca Leff, Awais Farhad, Kyle Pires, Muhammad Alamgir Khan, Lauren Hartz, Basmah Safdar

**Affiliations:** 1 Department of Emergency Medicine Yale School of Medicine New Haven, CT United States; 2 Department of Emergency Medicine Stanford School of Medicine Palo Alto, CA United States; 3 Department of Medicine Aga Khan University Karachi Pakistan; 4 CITRIC Health Data Science Center Aga Khan University Karachi Pakistan; 5 Department of Emergency Medicine Mayo Clinic School of Graduate Medical Education Rochester, MN United States; 6 School of Medicine Aga Khan University Karachi Pakistan

**Keywords:** natural language processing, gender-based differences, acute coronary syndrome, global health, Pakistan, gender, data, dataset, clinical, research, management, patient, medication, women, tool

## Abstract

**Background:**

Ischemic heart disease is a leading cause of death globally with a disproportionate burden in low- and middle-income countries (LMICs). Natural language processing (NLP) allows for data enrichment in large datasets to facilitate key clinical research. We used NLP to assess gender differences in symptoms and management of patients hospitalized with acute myocardial infarction (AMI) at Aga Khan University Hospital-Pakistan.

**Objective:**

The primary objective of this study was to use NLP to assess gender differences in the symptoms and management of patients hospitalized with AMI at a tertiary care hospital in Pakistan.

**Methods:**

We developed an NLP-based methodology to extract AMI symptoms and medications from 5358 discharge summaries spanning the years 1988 to 2018. This dataset included patients admitted and discharged between January 1, 1988, and December 31, 2018, who were older than 18 years with a primary discharge diagnosis of AMI (using *ICD-9* [*International Classification of Diseases, Ninth Revision*], diagnostic codes). The methodology used a fuzzy keyword-matching algorithm to extract AMI symptoms from the discharge summaries automatically. It first preprocesses the free text within the discharge summaries to extract passages indicating the presenting symptoms. Then, it applies fuzzy matching techniques to identify relevant keywords or phrases indicative of AMI symptoms, incorporating negation handling to minimize false positives. After manually reviewing the quality of extracted symptoms in a subset of discharge summaries through preliminary experiments, a similarity threshold of 80% was determined.

**Results:**

Among 1769 women and 3589 men with AMI, women had higher odds of presenting with shortness of breath (odds ratio [OR] 1.46, 95% CI 1.26-1.70) and lower odds of presenting with chest pain (OR 0.65, 95% CI 0.55-0.75), even after adjustment for diabetes and age. Presentation with abdominal pain, nausea, or vomiting was much less frequent but consistently more common in women (*P*<.001). “Ghabrahat,” a culturally distinct term for a feeling of impending doom was used by 5.09% of women and 3.69% of men as presenting symptom for AMI (*P*=.06). First-line medication prescription (statin and β-blockers) was lower in women: women had nearly 30% lower odds (OR 0.71, 95% CI 0.57-0.90) of being prescribed statins, and they had 40% lower odds (OR 0.67, 95% CI 0.57-0.78) of being prescribed β-blockers.

**Conclusions:**

Gender-based differences in clinical presentation and medication management were demonstrated in patients with AMI at a tertiary care hospital in Pakistan. The use of NLP for the identification of culturally nuanced clinical characteristics and management is feasible in LMICs and could be used as a tool to understand gender disparities and address key clinical priorities in LMICs.

## Introduction

Cardiovascular diseases (CVDs) represent a leading cause of mortality globally, with 80% of CVD deaths occurring in low- and middle-income countries (LMICs) [1,2]. Ischemic heart disease (IHD) is a leading cause of CVD death globally with a disproportionate burden borne by LMICs, due to a sharp rise in IHD risk factors, including increasing prevalence of obesity, aging of the population, and uncontrolled hypertension [3,4]. Pakistan is a LMIC with a population of over 200 million people [5], where IHD and associated IHD risk factors are on the rise, due to an ongoing epidemiological transition [6]. According to data from the Centers for Disease Control and Prevention, IHD is now the leading cause of death in Pakistan, surpassing diarrheal diseases, lower respiratory infections, and tuberculosis [7]. Consequently, there exists a high demand for novel approaches to further evaluate the epidemiological burden, clinical presentation, and adherence to medication guidelines for IHDs in Pakistan and other LMIC countries, but evidence-based approaches are lagging.

This paucity of data is particularly apparent in the evaluation of gender differences in the clinical presentation and medical management of IHD in LMICs [8,9]. A promising direction is the use of electronic health records (EHRs) to analyze patient data to better inform clinical decision-making and assess adherence to IHD treatment guidelines using a gender lens. However, the wealth of existing data in clinical records is generally inaccessible in LMICs as it exists in the form of free-text clinical narratives, as patient discharge summaries and hospital admission notes, and these are often handwritten [10,11]. Machine-learning tools stand to improve capacity in such resource-limited settings where the machines may be able to perform tasks that would otherwise be conducted by humans such as manual extraction of information from records. Natural language processing (NLP), a method by which algorithms permit machines to identify keywords and phrases in natural language corpora, has the potential to bypass human resources and other limitations, including the mining of unstructured textual data [10-12]. Methods based on analysis of EHRs through NLP algorithms have shown accuracy and precision in identifying patients at risk of health disparities presenting to health care providers in high-income countries [13-15], as well as in aiding in identifying IHD symptomatology from EHR records [16,17]. Only few studies exist evaluating the use of NLP in clinical contexts in LMICs, and even fewer have evaluated IHD [18-20].

To better understand the applications of NLP in this context, this study sought to use NLP to assess gender differences in symptoms and prescription of evidence-based medical therapies for patients hospitalized with acute myocardial infarction (AMI) at Aga Khan University Hospital (AKUH) in Karachi, Pakistan, using a previously curated dataset of discharge summaries. For the sake of improving readability and given two-thirds of the data described differences in symptom and management in gendered terms (as opposed to biological differences determined by patient “sex”), we have chosen the use of gender throughout this paper. In sum, we cannot determine the genetic sex of respondents in our study based on AMI clinical medical record data alone; therefore, gender is a more appropriate term to refer to self-identified or recorded societal representation in our study. To our knowledge, this will be the first study implementing NLP in a clinical context in Pakistan.

## Methods

### Study Site

Our data is derived from the AKUH, a leading tertiary academic hospital linked to 4 secondary hospitals based in Pakistan that treats approximately one million patients each year. Through a university-wide data initiative, coauthor ZS and team have developed processes to link and harness large datasets from the EHRs maintained in the health information management systems at AKUH.

### Dataset Characteristics

#### Overview

We used a previously curated data set that contained information on index admissions of patients with a primary diagnosis of AMI hospitalized at AKUH; this dataset included patients admitted and discharged between January 1, 1988, and December 31, 2018, who were older than 18 years with a primary discharge diagnosis of AMI (using *ICD-9* [*International Classification of Diseases, Ninth Revision*] diagnostic codes) [21]. For our study, we included patients from 2010 to 2018 as electronic copies of discharge summaries, readily available for that period (5358/5418 discharge summaries from that period were analyzed; n=60 discharge summaries were inaccessible). Extracted data from the EHRs comprised of admission and patient metadata and the discharge summaries. All data was deidentified before analysis, regarding privacy and confidentiality in this research.

#### Discharge Summaries

Each discharge summary extracted was in PDF format, containing both paragraphs of text (such as describing the patient history, a limited physical exam, the assessment, plan, and patient management during hospitalization) as well as tables. While some tables had completely structured information, others had free text in the cells. This study focused on 2 major categories of information from the discharge summaries: presenting symptoms and discharge medications.

Presenting symptoms varied in availability and structure across summaries. Summaries with symptoms lacked uniform headings and formats. Symptoms in tables were either occupying individual cells or punctuated into groups. Most summaries expressed symptoms in free text, noting patient presentation details or complaints. Such occurrences often appeared toward the beginning of the document. Occasionally, the middle of the summary commented on subsiding symptoms.

Discharge medication details consistently appeared toward the conclusion of each summary in tabular format, comprising structured information with a comprehensive list of both active and new medication prescriptions for the patient.

#### Admission Metadata

Metadata associated with the summaries, including patient gender, age at admission, admission year, medical comorbidities, and complications, was retrieved from the existing EHR system. These data were collated and stored in a dedicated spreadsheet.

### Model Development for Information Extraction

#### Overview

For this study, two different extraction models were created: the presenting symptom extraction model and the discharge medication extraction model.

#### Presenting Symptom Extraction Model

The primary aim of the presenting symptom extraction model was to identify specific keywords or phrases indicative of AMI symptoms within the discharge summaries.

The model first extracted the passage containing presenting complaints from discharge summaries using pattern-matching techniques. It then applied fuzzy keyword matching using the FuzzyWuzzy Python (Python Software Foundation) library to compare each word of the passage against the predefined list of keywords representing AMI symptoms. The list of AMI symptoms was initially compiled from UpToDate and Medical Subject Headings (National Institutes of Health) synonyms: UpToDate is a regularly updated electronic, clinical resource tool that is widely used by both physicians and patients. It provides information on clinical background, medication, preventative health measures, and other evidence-based clinical guidance. Medical Subject Headings terms are “hierarchically-organized vocabulary produced by the National Library of Medicine” that can be used for literature search purposes including for clinical research as in our case [22,23]. Afterward, this original list of AMI symptom terms underwent refinement by our team of local and international experts to improve contextual accuracy.

The presenting symptom extraction model used widely used fuzzy matching techniques [24-26] to account for variations in spelling, typos, and minor discrepancies between keywords and text in the presenting complaint passage. After manually reviewing the quality of extracted symptoms in a subset of discharge summaries through preliminary experiments, a similarity threshold of 80% was determined. This threshold indicates that if the text similarity between a word present in the patient’s complaint and the AMI symptom keyword exceeds 80%, the corresponding symptom is considered present in the patient’s complaint. Furthermore, to enhance accuracy, the model incorporated negation handling. It identified instances where negation words (such as “no” or “not”) occurred within a certain proximity to the matched keywords, thereby minimizing false positives.

#### Medication Extraction Model

Identification of tables containing discharge medication data was facilitated through the use of key indicator terms. Medication names used included medication brand names (such as Eziday and Telsan) and generic names (such as enalapril and amiodarone). Initial medication classification relied on categories from the Anatomical Therapeutic Chemical medication classification system, an internationally accepted standardized guideline for medication groupings [27]. For the classification of medications not available in this established dictionary, regular expressions were used to extract base terms from medication names. These terms were subsequently subjected to manual classification by our team of experts. The process of medication classification entailed the use of diverse pattern recognition techniques, aimed at reducing the necessity for manual labeling of medications into distinct classes.

For those discharge summaries included in the analysis, the frequency and proportion of medication classes and their combinations were obtained.

### Statistical Analyses

#### Overview

**Figure 1:**
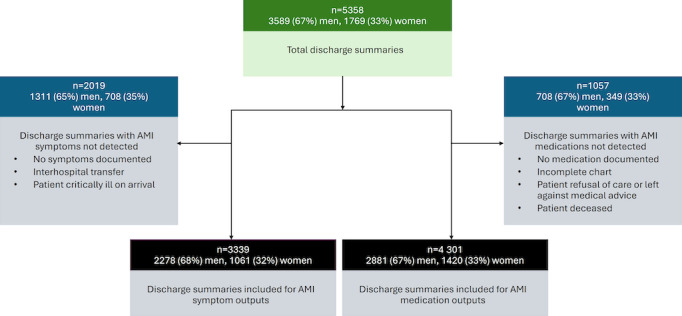
Schematic on model development and discharge summaries used for model outputs in a gender-based assessment of differences in acute myocardial infarction presentation and management using NLP in a dataset at Aga Khan University Hospital-Pakistan between 1998 and 2018. AMI: acute myocardial infarction; NLP: natural language processing.

The total number of discharge summaries evaluated for the two components of the model is outlined in Figure 1. Further, 2019 discharge summaries showed no AMI symptoms at the time of presentation to the hospital, and 1057 discharge summaries showed no detected AMI medications prescribed at the time of discharge from the hospital (Figure 1).

**Figure 2:**
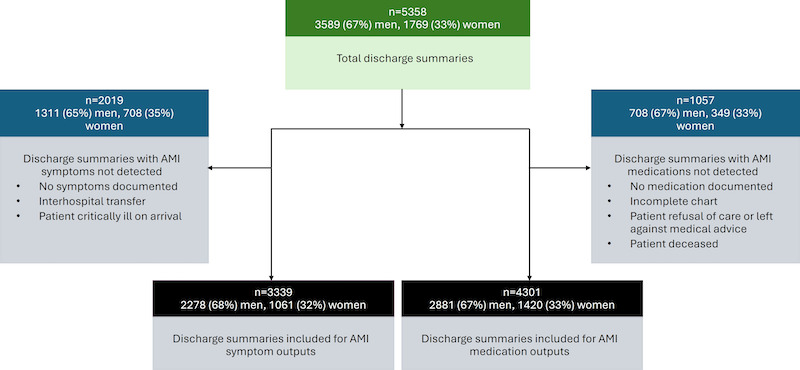
Updated Figure 2.

#### Independent Variables

The sociodemographic factor of gender was the sole independent variable assessed in this study. Gender was obtained from the EMR records associated with the patient files.

#### Dependent Variables

The main outcomes, or dependent variables of interest, for this study were clinical symptoms and medication prescription class. These variables were extracted from the dataset as noted above in the “extraction models.”

### Analysis

For descriptive analyses, frequency and proportions are presented. For symptom presentation, we conducted regression analyses controlling for age (comparing those younger than 45 y and those older than 45 y) hypothesizing based on well-established evidence that clinical presentation and risk factor profile differ distinctly between these age groups. For example, women have been found to experience a lack of chest pain—a distinguishing symptom in the diagnosis of AMI—at increasing rates for those aged younger than 45 years [28,29]. We also assessed symptom differences controlling for diabetes given variations in clinical presentation and potential management inherent to those with coafflictions of disease [30]. For medication prescription, we controlled for diabetic status as well as percutaneous intervention being conducted during hospital stay as we hypothesized that these would affect clinical management and prescription medications at discharge [31]. We also controlled for admission year to account for potential changes in the burden of disease, facility capacity, or clinical practice guidelines that may have occurred during the extent of the data collection period and that might affect both the likelihood of patient presentation and clinical practice or management. We then evaluated gender differences in symptoms and medication use from model outputs using chi-square or Mann-Whitney tests as appropriate. Both unadjusted and adjusted odds ratios (OR) are presented with associated 95% CIs.

### Role of the Funding Source

The funders of this study had no role in study design, data collection, data analysis, data interpretation, or writing of the report.

### Ethical Considerations

This research was determined to be exempt by the Yale Institutional Review Board as “not human subjects research” (2000026983). It was not appropriate or possible to involve patients or the public in the design, conduct, reporting, or dissemination plans of our research. This exemption is per the ethical standards of the responsible committee on human experimentation (institutional and national) and with the Helsinki Declaration of 1975.

## Results

### Overview

In our study, we analyzed a total of 5358 discharge summaries, comprising 1769 women and 3589 men. The mean ages of the patients included in the dataset between 2010 to 2018 (n=5358), were 67.8 years for women and 63.3 years for men.

### Gender Differences in AMI Presentation

Combining structured and unstructured data, as described in the Methods section, AMI symptoms were detected in 3339 discharge summaries, and they were not detected in 2019 charts as described in methods (eg, due to no IHD symptoms being documented, interhospital transfer, or being critically ill on arrival). Among the 3339 patient discharge summaries with symptoms described, chest pain and shortness of breath were the predominant presenting symptoms of AMI (Table 1). Chest pain was more common in men (56.27% of women and 68.79% of men, *P*<.001) as was diaphoresis (5.47% of women and 7.33% of men, *P*=.05), differences that persisted even after regression analysis (*P*<.001 and .06, respectively).

Shortness of breath was detected in 41.45% (1384/3339) of patients and found to be more common among women (49.48% of women and 37.71% of men, *P*<.001). Symptoms considered “atypical” for AMI—or termed “noncardiac” by 2021 AHA guidelines—such as abdominal pain (7.82% of women and 4.7% of men, *P*<.001), nausea or vomiting (9.9% of women and 5.44% of men, *P*<.001), and weakness (3.58% of women and 2.5% of men, *P*=.08) were also more likely to be reported among women [32]. These differences persisted even after controlling for diabetes and age (Table 2), except in the case of weakness. On subanalysis, comparing people with diabetes to people without diabetes, there were statistically higher odds of weakness in people with diabetes as compared to people without diabetes (63/95 or 3.45% of people with diabetes as compared to 32/95 or 2.11% of people without diabetes, *P*=.03).

“Ghabrahat,” a culturally distinct term best translated as *a sense of impending doom*, was detected in 4.1% (138/3339) of charts, with a trend for higher prevalence in women (3.69% of men and 5.09% of women, *P*=.06), although not statistically significant.

**Table 1:** Gender differences in frequency of symptom presentation detected in discharge summaries from a gender-based assessment on acute myocardial infarction presentation and management using natural language processing in a dataset at Aga Khan University Hospital-Pakistan between 1998 and 2018.

Symptom	Total cases (n=3339), n (%)	Women (n=1061, 32%), n (%)	Men (n=2278, 68%), n (%)	*P* value
Chest pain	2164 (64.81)	597 (56.27)	1567 (68.79)	<.001
Shortness of breath	1384 (41.45)	525 (49.48)	859 (37.71)	<.001
Nausea or vomiting	229 (6.86)	105 (9.9)	124 (5.44)	<.001
Sweating or diaphoresis	225 (6.74)	58 (5.47)	167 (7.33)	.05
Abdominal pain or discomfort	190 (5.69)	83 (7.82)	107 (4.7)	<.001
Arm pain	127 (3.8)	36 (3.39)	91 (3.99)	.40
Cultural term—“ghabrahat”	138 (4.13)	54 (5.09)	84 (3.69)	.06
Weakness	95 (2.85)	38 (3.58)	57 (2.5)	.08
Shoulder pain	93 (2.79)	30 (2.83)	63 (2.77)	.92
Palpitations	71 (2.13)	28 (2.64)	43 (1.89)	.16
Back pain	59 (1.77)	23 (2.17)	36 (1.58)	.23
Altered mental status	57 (1.71)	15 (1.41)	42 (1.84)	.37
Dizziness	43 (1.29)	14 (1.32)	29 (1.27)	.91
Jaw pain	29 (0.87)	15 (1.41)	14 (0.61)	.02
Orthopnea	32 (0.96)	14 (1.32)	18 (0.79)	.15
Neck pain	16 (0.48)	7 (0.66)	9 (0.4)	.31
Throat pain	4 (0.12)	0 (0)	4 (0.18)	—^a^
Swelling	1 (0.03)	1 (0.09)	0 (0)	—

^a^Not applicable.

**Table 2:** Adjusted odds of gender differences in symptom presentation detected in discharge summaries from a gender-based assessment on acute myocardial infarction presentation and management using natural language processing in a dataset at Aga Khan University Hospital-Pakistan between 1998 and 2018.

Symptom	Total, n (%)	Univariate		Multivariable^a^	
		OR^b^ (95% CI)	*P* value	OR (95% CI)	*P* value
Chest pain	2164 (64.8)	0.58 (0.5-0.68)	<.001	0.65 (0.55-0.75)	<.001
Shortness of breath	1384 (41.4)	1.62 (1.4-1.87)	<.001	1.46 (1.26-1.7)	<.001
Nausea or vomiting	229 (6.9)	1.91 (1.46-2.5)	<.001	1.85 (1.4-2.43)	<.001
Sweating or diaphoresis	225 (6.7)	0.73 (0.54-0.99)	.05	0.74 (0.55-1.02)	.06
Abdominal pain or discomfort	190 (5.7)	1.72 (1.28-2.32)	<.001	1.68 (1.24-2.26)	<.001
Arm pain	127 (3.8)	0.84 (0.57-1.25)	.40	0.91 (0.61-1.35)	.63
Cultural term—“Ghabrahat”	138 (4.1)	1.4 (0.99-1.99)	.06	1.36 (0.95-1.93)	.09
Weakness	95 (2.8)	1.45 (0.95-2.2)	.08	1.33 (0.88-2.03)	.18
Shoulder pain	93 (2.8)	1.02 (0.66-1.59)	.92	1.07 (0.68-1.67)	.77
Palpitations	71 (2.1)	1.41 (0.87-2.28)	.16	1.5 (0.92-2.45)	.10
Back pain	59 (1.8)	1.38 (0.81-2.34)	.23	1.39 (0.81-2.37)	.23
Altered mental status	57 (1.7)	0.76 (0.42-1.38)	.37	0.77 (0.42-1.4)	.39
Dizziness	43 (1.3)	1.04 (0.55-1.97)	.91	0.99 (0.52-1.9)	.98
Jaw pain	29 (0.9)	2.32 (1.12-4.82)	.02	2.1 (1.01-4.4)	.05
Orthopnea	32 (1)	1.68 (0.83-3.39)	.15	1.44 (0.71-2.92)	.31
Neck pain	16 (0.5)	1.67 (0.62-4.51)	.31	1.62 (0.6-4.38)	.34
Throat pain	4 (0.1)	Omitted	—^c^	Omitted	—
Swelling	1 (0)	Omitted	—	Omitted	—

^a^The multivariable model for each outcome (symptoms) was adjusted for diabetic status and age.

^b^OR: odds ratio.

^c^Not available.

### Gender Differences in Discharge Medications Among AMI Presentations

We assessed medication prescriptions among a total of 4301 records that were detected in discharge summaries by the model (Figure 1); 1057 records had no AMI medication prescriptions detected. We found a total of 9 different classes of medications (Table 3) after categorizing all individual medications into these classes a priori. The most common medication prescribed overall was aspirin (n=4070, 94.6%). This was followed by statins (n=3962, 92.1%), clopidogrel (n=3659, 85.1%), and β-blockers (n=3405, 79.2%). In total, 38 patients were prescribed a clopidogrel or aspirin combination pill. Nearly half of all patients were prescribed an antidiabetic (1795/4301, 41.7%) and around a third were prescribed an ace inhibitor (1685/4301, 39.2%).

Statistically significant gender differences in medication prescriptions were detected among all medications and classes except aspirin, aspirin or clopidogrel combinations, and ticagrelor (Table 3). Prescriptions for β-blockers, clopidogrel, statins, and ace-inhibitors were more common for men in univariate analysis. Those for antidiabetics and angiotensin receptor blockers (ARBs) were more common for women. For three of four first-line medications (clopidogrel, statin, or β-blocker), women were less likely than men to be prescribed medication, in univariate analysis. In multivariable analysis, statistically significant differences persisted for the first-line medications: statins, β-blockers, and ARBs after adjusting for diabetic status, year of admission, and percutaneous intervention. Women had nearly 30% lower odds (OR 0.71, 95% CI 0.57-0.90) of being prescribed statins, and they had 40% lower odds (OR 0.67, 95% CI 0.57-0.78) of being prescribed β-blockers.

**Table 3:** Odds of women being prescribed medication per discharge summaries from a gender-based assessment on acute myocardial infarction presentation and management using natural language processing in a dataset at Aga Khan University Hospital-Pakistan between 1998 and 2018.

Medication prescribed	Frequency and proportion of individuals who were prescribed medication (n=4301), n (%)	Univariate		Multivariable^a^	
		OR^b^ (95% CI)	*P* value	OR (95% CI)	*P* value
Aspirin	4070 (94.6)	0.86 (0.65-1.13)	.27	1.08 (0.82-1.44)	.57
Statin	3962 (92.1)	0.62 (0.49-0.77)	<.001	0.71 (0.57-0.9)	.004
Clopidogrel	3659 (85.1)	0.69 (0.58-0.82)	<.001	0.92 (0.76-1.11)	.39
β-blocker	3405 (79.2)	0.62 (0.54-0.72)	<.001	0.67 (0.57-0.78)	<.001
Antidiabetic	1795 (41.7)	1.51 (1.33-1.72)	<.001	1.08 (0.9-1.29)	.43
Ace-inhibitors	1685 (39.2)	0.75 (0.65-0.85)	<.001	0.91 (0.79-1.04)	.17
Angiotensin receptor blocker	546 (12.7)	1.64 (1.36-1.97)	<.001	1.64 (1.36-1.97)	<.001
Aspirin and clopidogrel combination	38 (0.9)	1.19 (0.61-2.3)	.61	0.91 (0.47-1.78)	.79
Ticagrelor	3 (0.1)	4.06 (0.37-44.83)	.25	3.44 (0.31-38.23)	.31

^a^The multivariable model for each outcome (medication) was adjusted for diabetic status, percutaneous intervention, and year of admission.

^b^OR: odds ratio.

## Discussion

### Principal Findings

In this study, we present the novel development of an NLP tool to detect gender differences in symptom presentation and medication classifications in a dataset of patients hospitalized with myocardial infarction at AKUH in Karachi, Pakistan. The data used in our study is a subset of the dataset restricted to the last decade when a discharge summary module was incorporated into the electronic medical record system (2008-2018). To the best of our knowledge, this is the first creation of an NLP model concerning IHD in Pakistan and appears to be the first health care application of NLP in the country. We demonstrated the effective design of such a model based on an unsupervised fuzzy string-matching technique using clinical and context-specific expertise.

There is a paucity of publications using NLP in the South Asia region [11,18,33], and even fewer that applied NLP clinically in the setting of an LMIC [11,18,20,34,35]. The first report using NLP in India was published in 2016 [11]. Literature is even more sparse for reports of IHD in LMICs [18-20]. The first annotated corpus addressing CVD risk factors using data from Chinese electronic medical records was built in 2017 [20]. The corpus was constructed with the help of clinicians and linguists. Our study adds to this study by applying NLP based approach for information extraction from medical records. Further, it extended the application to address IHD data in LMICs. Promoting the expansion and usage of NLP has the potential to advance the recognition of culturally specific nuances in a variety of disease states.

It is important to highlight gender, race, and cultural nuances in the symptom presentation of AMI to improve outcomes because delays have been associated with worse morbidity and mortality in several populations [36]. Although chest pain or discomfort is the most common symptom for both men and women with AMI, there are differences in the description of the symptoms, as well as several presenting symptoms that often lead to delays at both patient as well as provider levels. The classic description of AMI described as the “Levine sign” includes chest pain, difficulty breathing, sweatiness (diaphoresis), and possible radiation to the jaw, neck, or left arm [37]. Interestingly, we found the predominant symptom for AMI was also chest pain in the Pakistani cohort as has been reported in Western cohorts, although a third of the patients did not present with chest pain [32]. These numbers are similar to those reported in a large registry of patients with AMI in the United States which reported a third of patients with AMI present without chest pain and were associated with worse outcomes [29]. Instead, we found a high prevalence of “atypical” presentations including shortness of breath, weakness, nausea, and vomiting—symptoms not classically thought to be associated with AMI nor are they taught to lay people seeking care for these complaints. Similar to the US data, we found nonchest pain symptoms to be more prevalent among women with AMI as compared to men in the Pakistani population. Our study supports focusing on discrete symptoms and moving away from the description of AMI presentation from “typical” and “atypical” symptoms to reduce gender and racial disparities. Our message is consistent with the recent 2021 society guidelines for chest pain evaluation [32].

Our gender-specific data adds to the limited literature describing characteristics of AMI in the South Asian population, a cohort at particularly high risk for adverse cardiac outcomes and where most reports are limited to types of MI, comorbidities, and procedural details without providing information on the presenting symptoms [38-40]. The few that do list symptoms do not report them by patient gender. The gap is likely due to the exhaustive work of medical record reviews that is required to abstract symptoms, as well as an overall lack of gender-based research. Our model provides an example of how NLP can fill gaps in unharmonized free text information available through medical record reviews. Not only does this add to the literature by the variants (or commonalities) we found, but also provides a unique tool for use in similar large datasets to detect gender differences, as well as other underinvestigated populations.

Our study further adds to the existing literature on LMICs by describing the presence of variations in context-specific terms such as “ghabrahat.” Specifically, the term “ghabrahat” is an Urdu term to imply “impending doom” and is otherwise quite nonspecific for AMI. It was documented in 4.1% of the sample population, with a trend toward being reported more by women. This finding is important for both public health messaging as well as to guide clinicians on the importance of such unconventional but contextually specific terms in patients with AMI in the Pakistani setting. It also demonstrates the importance of research innovation in LMIC settings where clinical outcomes will affect the specific settings where studies are being conducted.

We used NLP to also describe gender-specific differences in AMI management in Pakistan, as indicated by medication prescription. The most common medications prescribed were aspirin, statins, and clopidogrel but were uniformly underused compared to guideline-based recommendations for AMI care. This underuse was even more pronounced in women when compared to men. Only ARBs were more commonly prescribed in women, which we posit may be a reflection of a higher prevalence of heart failure. Both statins and β-blockers were more commonly prescribed for men. Similar disparities in treatment based on gender have been noted in previous studies [41,42]. Possible explanations for these differences are gender differences with women presenting less frequently with classic chest pain, higher burden of comorbidities in women, and later age at presentation for women presenting with AMI as compared to men. Sex differences in the pathophysiology of AMI, with a higher proportion of myocardial infarction without obstructive coronary arteries, could also contribute to this disparity, although not directly measured in our data [43]. Future iterative improvement can be carried out by our NLP tool to detect comorbidities and complications and for the detection of patients being undertreated. Considering that these classes of medications are also used in other CVDs such as stroke, our model can be generalized to assess medication prescription patterns in other diseases.

### Limitations

Our study has several limitations. The development of the model may have been subject to observation or confirmation bias based on individual biases among members of the research team. However, we feel this is unlikely to have affected the quality and effectiveness of the model, as we had purposeful diverse representation in the team. As mentioned previously, this included experts in cardiology, emergency medicine, global health, informatics, as well as representation from the consultant, fellow, resident, and medical student level, and equitable representation by gender. Moreover, there was adequate representation in our team from the study site (AKUH, Pakistan) to provide overall guidance, cross-cultural translation of concepts, and context-specific nuances during the development and validation of the tool. We feel that the rigor and diversity of the team contributed to the overall high accuracy of the model. In the future, we plan to test the model among patients with IHD in similar settings.

Naturally, due to missing data such as from incomplete charts or deceased patients discussed in methods (Figure 1), our findings are at risk for selection bias. However, the output generated by the extraction models was manually reviewed on randomly selected summaries to mitigate selection bias. Moreover, our findings are ratified by existing evidence on gender disparities in patients with IHD. Future studies may also promote the development of models that assess additional free-text clinical narratives in the medical record, drawing from triage and admission notes to provide a more comprehensive assessment of patient presentation that may be available in these sources. However, in light of these limitations, we provide compelling evidence that NLP is a valid option for harnessing data outputs from large datasets in the clinical setting from this region.

Furthermore, our study also lacks traditional expert medical record review as a reference standard for validating the proposed NLP methodology based on the fuzzy string-matching technique used to extract presenting AMI symptoms. Instead, we manually reviewed a subset of the extracted data to confirm the accuracy of the presenting symptom extraction model. This limitation highlights an area for future research, where further validation through expert review could improve the robustness of our findings.

### Conclusions

In conclusion, the use of NLP for the identification of clinical characteristics and management of IHD is plausible in LMICs. Diverse content and regional expertise, including local clinical experts, may contribute to effective tool development for context-specific settings. Using this tool, we identified gender differences in clinical symptomatology in Pakistani patients with AMI, with women more likely to present with nonchest pain symptoms. Furthermore, we also noted gender differences with fewer first-line prescriptions being provided to women. In summary, opportunities exist for the implementation of NLP to guide clinical practice in Pakistan, and similar LMIC settings.

## Abbreviations

AKUH: Aga Khan University Hospital
AMI: acute myocardial infarction
ARB: angiotensin receptor blocker
CVD: cardiovascular disease
EHR: electronic health record
ICD-9: International Classification of Diseases, Ninth Revision
IHD: ischemic heart disease
LMIC: low- and middle-income country
NLP: natural language processing
OR: odds ratio

## Data Availability

The data used for this analysis can be made available on reasonable request once all relevant substudies from the NLP in AMI presentation and management in this Pakistan study are reported and completed. The data dictionary can be made available on request to the corresponding author.
